# Multivalent Interaction of Beta-Catenin With its Intrinsically Disordered Binding Partner Adenomatous Polyposis Coli

**DOI:** 10.3389/fmolb.2022.896493

**Published:** 2022-06-08

**Authors:** Pamela J. E. Rowling, Ben L. Murton, Zhen Du, Laura S. Itzhaki

**Affiliations:** Department of Pharmacology, University of Cambridge, Cambridge, United Kingdom

**Keywords:** beta-catenin (β-catenin), adenomatous polyposis coli (APC), intrinsically disordered protein, protein-protein interaction (PPI), multivalency, fuzzy binding, armadillo repeat

## Abstract

The Wnt signalling pathway plays key roles in cell proliferation, differentiation and fate decisions in embryonic development and maintenance of adult tissues, and the twelve Armadillo (ARM) repeat-containing protein β-catenin acts as the signal transducer in this pathway. Here we investigate the interaction between β-catenin’s ARM repeat domain and the intrinsically disordered protein adenomatous polyposis coli (APC). APC is a giant multivalent scaffold that brings together the different components of the so-called “β-catenin destruction complex”, which drives β-catenin degradation *via* the ubiquitin-proteasome pathway. Mutations and truncations in APC, resulting in loss of APC function and hence elevated β-catenin levels and upregulation of Wnt signalling, are associated with numerous cancers including colorectal carcinomas. APC has a long intrinsically disordered region (IDR) that contains a series of 15-residue and 20-residue binding regions for β-catenin. Here we explore the multivalent nature of the interaction of β-catenin with the highest affinity APC repeat, both at equilibrium and under kinetic conditions. We use a combination of single-site substitutions, deletions and insertions to dissect the mechanism of molecular recognition and the roles of the three β-catenin-binding subdomains of APC.

## Introduction

**FIGURE 1:**
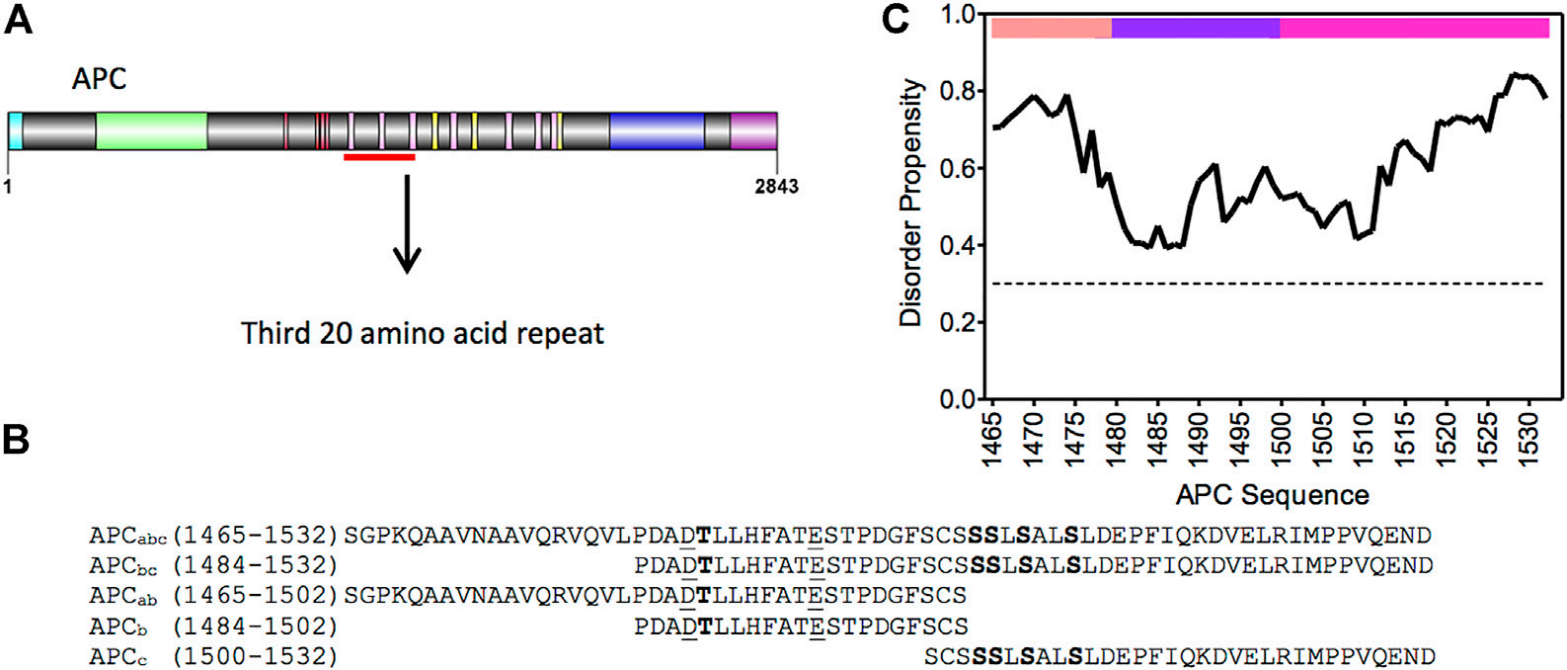
APC and β-catenin. **(A)** Schematic of the domain structure of APC from N- to C-terminus: coiled-coil oligomerisation domain, turquoise; armadillo domain, light green; β-catenin- binding 15aa repeats, red; β-catenin-binding 20aa repeats, pink; axin-binding SAMP repeats, yellow; microtubule-binding basic region, blue; and EB1-binding domain, purple. The mutational cluster region is underlined in red. **(B)** Sequence of the third 20aa repeat construct of APC, and fragments thereof, used in this study. Phosphorylated residues are in bold, and lysine-interacting residues are underlined. **(C)** Prediction of disorder propensity of the third 20aa repeat (R3) of APC using the flDPnn program. Amino acids with values above the dotted line are predicted to be disordered and those below ordered (Hu et al., 2021). The coloured bars at the top of the graph represent the three subdomains of APC R3: the N-terminal α-helical domain is in peach (APC_a_), the lysine-binding domain in purple (APC_b_), and the phosphorylation domain in pink (APC_c_).

**FIGURE 2:**
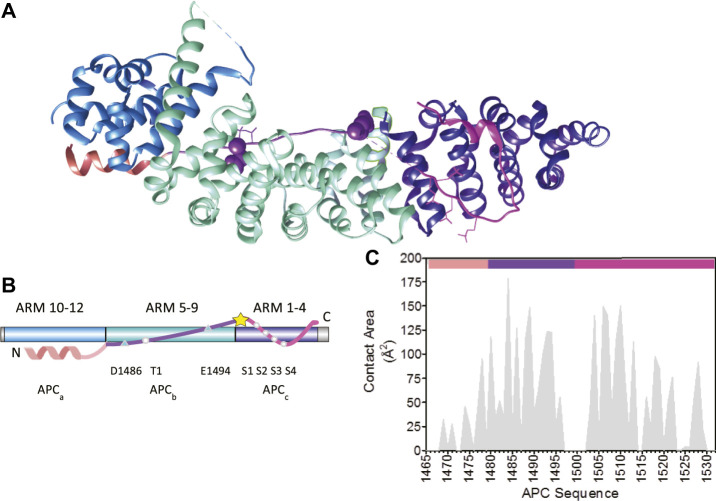
The interaction of the third 20aa repeat (R3) of APC with β-catenin. **(A)** Schematic representation of the structure of the armadillo repeat domain of β-catenin in complex with the phosphorylated third 20aa repeat of APC(R3) (PDB 1TH1) (Xing et al., 2004). The three subdomains of APC R3 are coloured: the N-terminal α-helical domain in peach (APC_a_), the lysine binding domain in purple (APC_b_), and the phosphorylation domain in pink (APC_c_). The amino acids that are phosphorylated are represented as sticks and the two lysine-interacting amino acids as spheres. The β-catenin ARM repeats that bind to the three APC subdomains are coloured: ARM 10–12 in blue, ARM 5–9 in turquoise, and ARM 1–4 in dark blue. **(B)** Schematic showing the location of the key β-catenin-binding residues in APC R3. APC and β-catenin are coloured as in **(A)**. The five phosphorylated residues, S1–S4 and T1, are represented by circles. The lysine-binding residues D1486 and E1494 are represented by triangles. The site of the single cysteine residue (C1501) is represented by a yellow star. **(C)** Buried surface area by residue of pAPC R3 in the complex with β-catenin derived from PDB 1TH1 calculated using Cocomaps (http://www.molnac.unisa.it/BioTools/cocomaps, Vangone et al., 2011). The bars at the top of the graph indicate the three subdomains of APC R3 coloured as in **(A)**.

The study of protein-protein interactions (PPI) is key to understanding protein functionality within a cell: signaling, transport, catalysis, etc. The majority of PPIs events involve the interactions of 10 or fewer amino acid between two binding partners. However, there are exceptions to this rule; one example being the interaction between adenomatous polyposis coli (APC) and β-catenin, which forms an unusually large and elongated binding surface with approximately 5000 A˚^2^ of surface area buried (Figures 1, 2) (Ha et al., 2004; Xing et al., 2004). APC is a multifunctional protein contributing to proliferation, differentiation and migration in cells by regulating the levels of β-catenin available for transcription of the LEF-TCF4 family of transcription factors and by controlling the stability of microtubules during interphase and mitosis. APC is a tumour suppressor gene, and alterations in the gene are an early event in 80%–85% of sporadic colorectal cancers and germline mutations that lead to colorectal cancer in familial adenomatous polyposis (McCartney and Näthke, 2008; Zhang and Shay, 2017; Caspi et al., 2021). APC is a 310 kD protein with a coiled-coil dimerization domain region at the N-terminus and an armadillo repeat domain that interacts with proteins involved in cell migration and adhesion; at the C-terminus are domains that bind to microtubules involved in APC functions in chromosomal and mitotic progression. The central region comprises approximately 1,000 amino acids and is predicted to be intrinsically disordered (Figure 1C). It contains eleven potential β-catenin binding sites consisting of four 15 amino acid (15aa) repeats and seven 20 amino acid (20aa) repeats, which can be phosphorylated, and three SAMP regions that bind axin (Figure 1A).

Tandem-repeat proteins such as β-catenin contain arrays of small structural motifs (20–40 amino acids) that pack in a linear fashion to produce elongated, one-dimensional architectures with a continuous hydrophobic core and extended solvent-accessible surfaces (Figure 2A) and are ubiquitous in nature, their chief function being to bind other proteins (Javadi and Itzhaki, 2013). β-catenin has a 530-residue central domain of 12 tandem armadillo (ARM) repeats (Huber et al., 1997; Xing et al., 2008), which are 42-residue motifs that form a triangle of three helices. The twelve imperfect ARM repeats of β-catenin stack linearly to form a right-handed superhelix of helices that can be divided into three regions: ARM repeats 1–4 to which axin binds, ARM repeats 5–9 which contains the groove used by a number of β-catenin binding partners, and the third region formed by ARM repeats 9–12. The third helix of each ARM repeat lines the groove formed by the superhelix and is enriched in positively charged residues creating an extended docking site shared by a number of β-catenin’s negatively charged intrinsically disordered binding partners including ICAT, APC, E-cadherin and TCF7/L2 (Eklof Spink et al., 2001; Poy et al., 2001; Graham et al., 2002; Xing et al., 2003; Ha et al., 2004) (Figure 1D). The highly elongated interface nature of the interfaces between natural ARM repeat proteins and IDRs is currently being exploited by Pluckthun and colleagues to build artificial ARM proteins capable of recognizing any linear peptide sequence (Hansen et al., 2016).

β-catenin carries out two distinct functions in the cell, the first of which is as the signal transducer in the canonical Wnt signalling pathway (Van Der Wal and Van Amerongen, 2020) and resulting in the transcription of genes that are of developmental importance and those involved in tissue homeostasis. In the absence of a Wnt signal, cytosolic β-catenin is continuously synthesised and then sequestered and targeted for proteasomal degradation by a multi-protein complex called the β-catenin destruction complex (BDC) that forms biological condensates (Schaefer et al., 2018). The BDC comprises five different proteins: two structural proteins, APC and axin, three kinases [glycogen synthase kinase 3β (GSK3β) and casein kinase 1α and casein kinase 1ε (CK1α, CK1ε)], and protein phosphatase 2A (PP2A). In the BDC β-catenin is hyperphosphorylated at its N-terminal disordered region by the combined action of GSK3β and CK1α (Kimelman and Xu 2006) and subsequently recognised and ubiquitinated by the E3 ubiquitin ligase β-TrCP (Wu et al., 2003) and degraded by the proteasome. The second function of β-catenin is as an adaptor mediating cell-cell adhesion at adherens junctions, whereby β-catenin binds to the intracellular domain of the cadherin family of proteins (Huber and Weis, 2001; Pokutta and Weis, 2007; Harris and Tepass, 2010; van der Wal and van Amerongen, 2020).

The behaviour of IDPs in complex with their binding partners can be divided into static complexes (Uversky, 2011), in which the IDP is ordered and hence visible in X-ray crystal structures), and dynamic or “fuzzy” complexes, in which the IDP retains a degree of disorder upon complex formation (Sigalov et al., 2007; Borg et al., 2007; Mittag et al., 2009). IDP binding mechanisms have been grouped into four classes: simple two-state binding, avidity, allovalency, and so-called “fuzzy” binding (Olsen et al., 2017), reflecting the variability in disorder and the extent to which the disorder and conformational heterogeneity is retained upon complex formation; even in the simple two-state case, association can involve conformational selection or folding upon binding (Wright and Dyson, 2009; Bachmann et al., 2011; Dogan et al., 2014; Rogers et al., 2014; Karlsson et al., 2020). Given the very long interfaces involved, the interaction of β-catenin with its binding partners provides a striking system with which to study IDR molecular recognition (Smith et al., 2021; Wiggers et al., 2021). In the case of APC, the intrinsically disordered 20aa repeats adopt an extended structure that wraps in an anti-parallel fashion around the entire ARM domain of β-catenin (Liu et al., 2006; Xue et al., 2012; Minde et al., 2013) (Figure 2). The 20aa repeats of APC contain a conserved core motif (SLSSLS); a similar motif is also found in the β-catenin-binding region of E-cadherin, and both APC and E-cadherin are phosphorylated by casein kinase 1 (CK1) and glycogen synthase kinase 3 (GSK3) (Ikeda et al., 2000; Rubinfeld et al., 2001), and binds ARM1-4 of β-catenin. Additionally, two negatively charged residues, Asp1486 and Glu1494 in the N-terminal region flanking the core form salt bridges with two lysine residues [K435 and K312, respectively (known as lysine “buttons”)] within ARM5-9 of β-catenin, and these lysine residues are also essential for the interaction of β-catenin with Tcf and E-cadherin. Lastly, an alpha-helical N-terminal region of the APC 20aa repeat interacts with ARM10-12. Strikingly, the individual 20aa repeats in APC vary greatly in their binding affinities, and it remains to be determined exactly how the repeats work together to regulate β-catenin in the cell (Liu et al., 2006; Seo and Jho, 2007; Su et al., 2008; Roberts et al., 2011). Here, using single-site substitutions, domain truncations and insertions, we dissect the contributions to β-catenin binding of the three different interfaces within the highest-affinity third 20aa repeat of APC (R3).

## Materials and Methods

### Molecular Biology and Protein Expression and Purification

The plasmids encoding the armadillo (ARM) repeat domain of human β-catenin (residues 134–671) and APC were kind gifts from Prof. W. I. Weis and Dr. S. H. McLaughlin, respectively. The R3 region of APC and fragments thereof (Figure 1) and shorter constructs of APC were produced by PCR, and mutations were introduced using site-directed mutagenesis. All plasmids used for expression were cloned into a modified pRSET vector where the His-tag has been replaced by a GST-tag and a thrombin cleavage site introduced between the GST and the protein of interest; after cleavage this leaves two amino acids, GS, at the N-terminus of the protein. All proteins were expressed in *E. coli* C41 cells (Miroux and Walker, 1996). Transformed cells were grown in 2TY media with appropriate antibiotic until OD600 of 0.6 at 37°C was reached, the temperature was then lowered to 25°C and the cells induced with 0.2 mM IPTG after a further 18 h the cells were harvested at 5,000 g for 7 min at 4°C. The cells were resuspended in 50 mM Tris-HCl buffer pH7.5, 150 mM NaCl, 1 mM DTT containing protease inhibitors and lysed using an Emusiflex C5 (Avestin) at 10,000 psi. The lysed cells were centrifuged at 35,000 g for 35 min at 4°C. The proteins were purified using glutathione-Sepharose 4B beads (Cytiva). The GST was cleaved from the target protein on the resin using thrombin and the protein eluted.

β-catenin was further purified using a Mono-Q column (Cytiva) equilibrated in 50 mM Tris-HCl buffer pH 8.9, 50 mM NaCl, 1 mM DTT and eluted with a linear NaCl gradient to 1M NaCl. Fractions containing greater than 95% β-catenin were pooled, flash frozen, lyophilised, and stored at −80°C (Supplementary Figure S1). Further purification of the APC constructs was achieved by removing high molecular weight impurities using differential filtration; the samples were centrifuged through a centrifugal concentrator with a 30 kD molecular weight cut-off (MWCO) membrane, and the flow through was concentrated using a 3 kD MWCO centrifugal concentrator (Supplementary Figure S2). The purified protein was then flash frozen and stored at −80°C. The identities of all proteins were confirmed by MALDI mass spectrometry performed by Dr. Len Packman (University of Cambridge PNAC Facility). β-catenin concentration was calculated from its extinction coefficient at 280 nm obtained from ProtParam (Gasteiger et al., 2005), and APC concentration was measured using the Pierce™ BCA protein Assay Kit (ThermoFisher).

### Labelling of APC With Fluorescent Tags

APC constructs were labelled at the single cysteine residue located at position 1,501, which was retained in all of the fragments made here. Proteins were buffered exchanged into PBS containing 1 mM TCEP and labelled with either fluorescein-maleimide or Alexa Fluor™ 488 C5 maleimide. Fluorescein-maleimide was added at a 5-fold molar excess concentration to protein and incubated for 1 h at room temperature; excess dye was removed by acetone precipitation of the APC construct (Vivès and Lebleu, 2003); the labelled protein was resuspended in PBS, 1 mM DTT. A 2-fold molar excess of Alexa Fluor™ 488 C5 maleimide was added to the protein for 2 h at room temperature and excess dye was removed using Pierce™ Dye Removal Columns (ThermoFisher). The labelling efficiency was determined from the ratio of the concentration of the fluorescein/Alexa Fluor™ 488 moiety to the protein concentration, and additionally the number of sites labelled per molecule was assessed by MALDI mass spectrometry (performed by Dr. Len Packman, University of Cambridge PNAC Facility). This analysis showed that all of the APC constructs were labelled at >90% and at the single intended site (Cysteine 1501). Labelled APC was flash frozen and stored at −80°C.

### Equilibrium Binding Measurements

The binding of fluorescent labelled APC constructs to β-catenin was monitored either by fluorescence or by fluorescence anisotropy using a LS55 fluorimeter (Perkin Elmer). All proteins were buffer exchanged into PBS, 1 mM DTT, and measurements were made at 25°C. Excitation and emission wavelengths were 495 and 519 nm, respectively, and slit widths were 5 nm. APC_bc_ labelled at Cys 1501 with Alexa-488 was used in a competition fluorescence assay to measure the IC_50_ values of the phosphomimetic APC_bc_ variants for β-catenin. In these experiments, a solution of 20 nM Alexa-488-APC and 200 nM β-catenin was incubated in the cuvette at 25°C for 30 min prior to titration of unlabelled APC_bc_ variants using a Microlab 500C dispenser (Hamilton). After addition of each aliquot, the sample was stirred for 30 s and equilibrated for a further 60 s before measurement. The IC_50_ was calculated using the following equation:

$$F=F_{f}+\frac{\text{Δ}F}{\left(1+\left(\frac{\left[U\right]}{IC_{50}}\right)\right)}$$ (1)

where *F* is the measured fluorescence, *F*_*f*_ is the fluorescence of the free ligand, Δ*F* is the change in fluorescence between the free and bound form, and *U* is the concentration of the competing APC ligand.

All other APC constructs were labelled with fluorescein, and the dissociation constants were measured by fluorescence anisotropy. In these experiments, aliquots of β-catenin were titrated into a cuvette containing 10 nM fluorescein-labelled APC, and the sample was stirred and equilibrated before measurement (as described above). Both fluorescence anisotropy and fluorescence intensity were measured at 15°C. As there was a difference in the fluorescence intensity of bound and unbound APC, the fluorescence anisotropy was adjusted using the following equation:

$$R_{adj}\;=\frac{\left(\frac{R-R_{f}}{R_{b}-R}\right)\left(\frac{Q_{f}}{Q_{b}}\right)R_{b}+R_{f}}{1+\left(\frac{R-R_{f}}{R_{b}-R}\right)\left(\frac{Q_{f}}{Q_{b}}\right)}$$ (2)

where *Q* is the measured fluorescence and *R* is the measured anisotropy. The subscripts *f* and *b* denote the unbound (free) and saturated bound forms of labelled APC. The adjusted anisotropy data were then fitted using a single-state Hill plot to derive the *K*_d_ and Hill coefficient, *h*:

$$R=\frac{R^{h}P^{h}}{K_{d}^{h}+P^{h}}$$ (3)

where *P* is the APC concentration

To calculate the Gibbs free energy of binding from the *K*_d_, Equation 4 was used:

$$\text{Δ}\mathrm{G=}RT\text{ ln }K_{\text{d}}$$ (4)

where *R* is the gas constant and T is the temperature in Kelvin.

### Kinetic Experiments

Stopped-flow fluorescence was used to measure the kinetics of binding with a SX-19 stopped-flow fluorimeter (Applied Photophysics). The excitation wavelength was 495 nm, and a cut-off filter of 515 nm was used to measure the emission. The slit widths were 2 nm for both excitation and emission. All proteins were prepared in PBS buffer, 1 mM DTT, and the experiments were performed at 15°C. The β-catenin concentrations used were at least ten times higher than that of the APC to ensure pseudo first-order conditions; a fixed concentration of fluorescein-labelled APC (25 nM) was rapidly mixed with varying concentrations of unlabelled β-catenin (between 250 and 2,000 nM) in a 1:1 volume ratio, and the change in fluorescence intensity was recorded. For dissociation experiments, the complex of β-catenin and labelled APC was pre-formed by mixing the two proteins in a 1:1 molar ratio (200 nM) and incubated for 1 h at 15°C in the dark. The pre-formed complex was then mixed in a 1:1 volume ratio with 10 times molar excess of the same APC construct, and the change in fluorescence intensity was measured. A minimum of five traces was collected and averaged. The averaged trace was plotted using GraphPad Prism 5 (GraphPad Software, Ltd.) and fitted to either a single exponential phase or the sum of two exponential phases. For the association experiments, the observed rate constant, *k*_*obs*_, was plotted against the concentration of β-catenin, and *k*_*on*_ was calculated from the linear fit:

$$k_{obs}=k_{on}\left[\text{β catenin}\right]+k_{off}$$ (5)

## Results

The region of APC used in this work encompasses the third 20aa repeat (R3) (residues 1,494–1,514) and flanking regions that contribute to the interaction with β-catenin (Figure 1). This repeat was shown previously to have the highest affinity for β-catenin of all APC repeats (Liu et al., 2006), and there are structures of both the phosphorylated repeat (Figure 1D) and the unphosphorylated repeat in complex with β-catenin (Ha et al., 2004; Xing et al., 2004; Choi et al., 2006). It can be subdivided into three subdomains in terms of its interaction with β-catenin: residues 1,465–1,483 (referred to subsequently as the helical domain “a”) that bind to ARM10-12 of β-catenin; residues 1,483–1,502 (the lysine-binding domain “b”) that bind to ARM5-9; and residues 1,502–1,532 (the phospho domain “c”) that bind to ARM1-4 (Figure 2).

### Effects of Phosphomimetic Substitutions in APC

**FIGURE 3:**
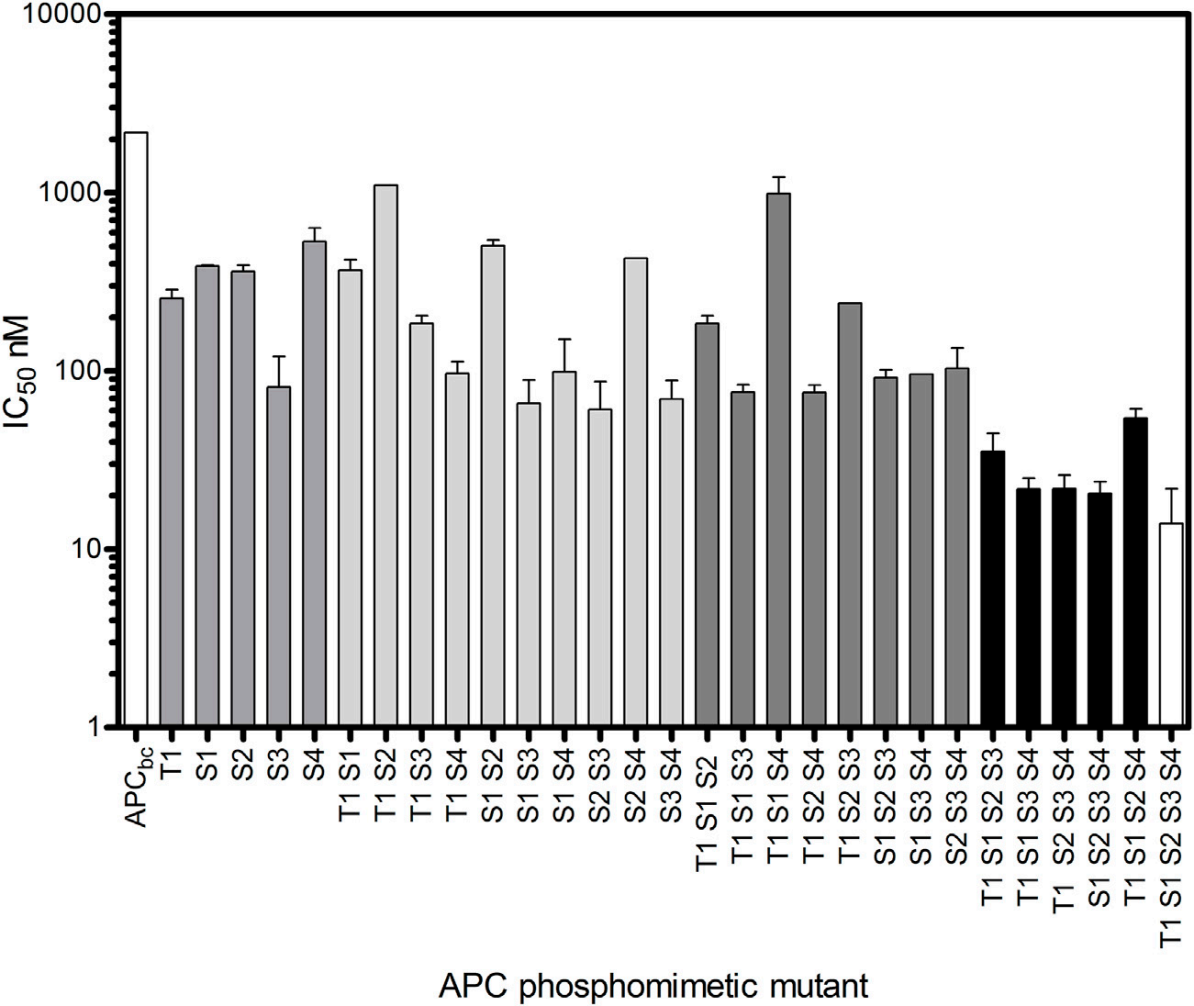
Increasing affinity for β-catenin of APC_bc_ constructs with increasing number of phosphomimetic substitutions. Glutamate substitutions were made at phosphorylation sites T1487E (T1), S1504E (S1), S1505E (S2), S1507E (S3), S1510E (S4). The IC_50_s of these phosphomimetic APC_**bc**_ variants was measured by fluorescence competition assay using APC_**bc**_ labelled with Alexa488 at Cys 1501. The data are shown as the mean ± SEM of three replicates.

It is important to note that in the crystal structures of β-catenin-APC and β-catenin-E-cadherin complexes, both of which show similar contacts between β-catenin and the phosphorylation sites of the respective ligands, there appears to be no interaction between β-catenin and these residues in their unphosphorylated forms (Huber and Weis, 2001; Ha et al., 2004). In addition to the β-catenin contacts made by the phosphorylated sites of APC spanning residues 1,487–1,510, there are other contacting residues, notably within 1,510–1,529 that folds back onto 1,487–1,510 (Figure 2). With an APC construct comprising subdomains b and c (residues 1,483–1,533), referred to as APC_bc_, the five phosphorylation sites were mutated to the phosphomimetic glutamate either individually or in combination [T1487E (T1), S1504E (S1), S1505E (S2), S1507E (S3), S1510E (S4)], and the IC50s were measured by competition fluorescence assay using APC_bc_ labelled with Alexa-488 at Cys 1,501 (Figure 3).

Mutation of any one of these residues to the phosphomimetic increases the affinity for β-catenin between 4-fold and 30-fold, with S1507E showing the greatest increase; in the crystal structure of the complex, phospho-S1507 (S3) is tightly coordinated and fixed in a specific orientation by three β-catenin residues and therefore would be predicted to have the tightest binding affinity of the five phosphorylated residues. Phospho-S1505 (S2) does not appear to make any contacts with β-catenin in the crystal structure of the complex, but the phosphomimetic mutation at this site nevertheless increased the binding affinity 5-fold. This could be as result of the interaction of E1505 with R1523 within the short helix formed by this region of APC, which could stabilise its interaction with β-catenin. Alternatively, it may reflect a “fuzzy” behaviour of the APC-β-catenin interaction, in which the interface, and the interactions formed by specific residues, in solution is different from the static structure captured in the crystallography. Constructs containing four or more phosphomimetics showed a ∼150-fold increase in affinity.

**FIGURE 4:**
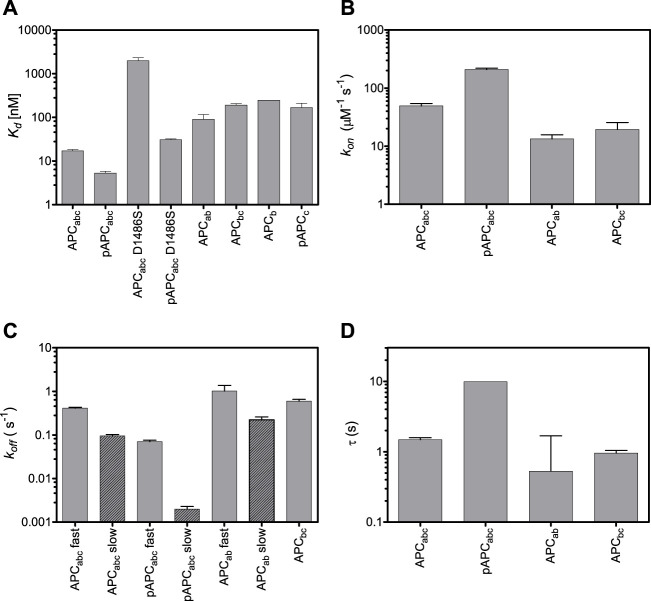
Effects of truncations and mutations in APC on β-catenin-binding. Dissociation constants **(A)**, on-rates **(B)**, off-rates **(C)**, and time constants **(D)**. Both fast and slow off-rates are shown. The time constant are calculated from the fast off-rates.

The affinity of the full-length R3 domain, APC_abc_ containing all five phosphomimetics (referred to subsequently as pAPC), was also measured by fluorescence anisotropy, (see next section), and the *K*_d_ determined to be 5.3 nM (Table 1; Figure 4). This value is similar to *K*_d_ values obtained in previous studies using an APC R3 construct that had been phosphorylated using CK1-ε and GSK3-β (Ha et al., 2004; Xing et al., 2004; Choi et al., 2006; Liu et al., 2006), indicating that glutamate serves as a good phosphomimetic.

**TABLE 1:** Effects of mutations, truncations and insertions in APC Repeat 3 region on the interaction with β-catenin. p-APC indicates phosphomimetic mutations, APC indicates non-phosphorylated form. Presence or deletion of subdomains a, b and c is indicated in subscript. FL indicates flexible linker, RL indicates rigid linker, PP indicates polyproline linker (see also Figure 5A), and the site of linker insertion is indicated in subscript. In the schematics, the key contacting residues are denoted in circles (phosphomimetics) and triangles (lysine-binding residues). ND indicates not detectable. The values of *K*_*d*_ *, ΔG* and Hill coefficient are derived from the fluorescence anisotropy experiments. The on- and off-rates and time constant (τ, calculated from the fast off-rates) were determined by stopped-flow fluorescence experiments. ND indicates not detectable. The SE error come from at least three replicates.

APC fragment	Schematic of APC R3 region	*K*_d_ (nM)	Δ*G* (kcal mol^−1^)	Hill coefficient	*k*_on_ (µM^−1^s^−1^)	*k*_off_ fast (s^−1^)	*k*_off_ slow (s^−1^)	τ (s)
APC_abc_	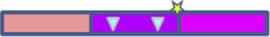	16.5 ± 5.1	−10.6 ± 0.1	1.09 ± 0.1	49.2 ± 5.1	0.46 ± 0.06	0.11 ± 0.02	1.54 ± 0.23
APC_ab_	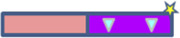	89.9 ± 26.6	−9.6 ± 0.2	1.3 ± 0.5	13.3 ± 1.7	1.12 ± 0.31	0.24 ± 0.032	0.66 ± 0.18
APC_bc_	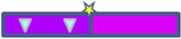	196 ± 18.7	−9.2 ± 0.1	1.1 ± 0.1	14.8 ± 1.5	0.73 ± 0.06	ND	0.96 ± 0.08
APC_b_	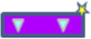	246 ± 3.5	−9.0 ± 0.1	0.9 ± 0.1	—	—	—	—
pAPC_abc_	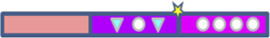	5.3 ± 0.6	−11.3 ± 0.1	2.0 ± 0.2	207 ± 11	0.07 ± 0.01	0.0021 ± 0.003	9.51 ± 0.53
pAPC_c_	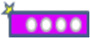	160 ± 37.0	−9.2 ± 0.2	1.1 ± 0.2	—	—	—	—
APC_abc_D1486S	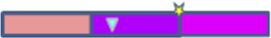	2054 ± 213	−7.8 ± 0.1	0.8 ± 0.1	ND	ND	ND	ND
pAPC_abc_D1486S	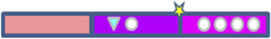	21.4 ± 1.2	−10.5 ± 0.1	1.2 ± 0.3	151 ± 14	2.89 ± 0.01	0.400 ± 0.002	0.24 ± 0.006
APC_abc_FL_1500_	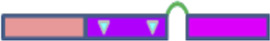	28.3 ± 5.9	−10.3 ± 0.1	1.0 ± 0.1	24.1 ± 7.0	0.45 ± 0.04	0.10 ± 0.003	1.54 ± 0.13
APC_abc_RL_1500_	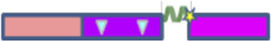	31.2 ± 3.6	−10.3 ± 0.1	0.9 ± 0.1	18.1 ± 1.6	0.89 ± 0.02	0.223 ± 0.002	0.80 ± 0.14
pAPC_abc_FL_1500_	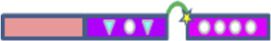	10.9 ± 2.4	−10.6 ± 0.1	1.8 ± 0.1	101.0 ± 9.6	0.16 ± 0.18	0.0085 ± 0.0015	4.45 ± 1.16
pAPC_abc_RL_1500_	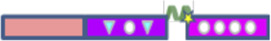	10.8 ± 2.9	−10.9 ± 0.2	2.0 ± 0.3	102.5 ± 7.9	0.21 ± 0.02	0.0122 ± 0.0016	3.52 ± 0.78
pAPC_abc_PP_1500_	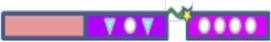	16.4 ± 2.5	−10.6 ± 0.1	1.2 ± 0.2	89.7 ± 5.1	0.44 ± 0.035	0.018 ± 0.002	1.59 ± 0.24
APC_abc_FL_1478_	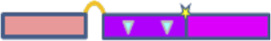	21.5 ± 3.5	−10.5 ± 0.1	1.4 ± 0.2	83.5 ± 2.4	0.77 ± 0.038	0.177 ± 0.007	0.90 ± 0.04
pAPC_abc_FL_1478_	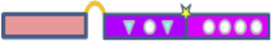	18.7 ± 0.17	−10.5 ± 0.1	1.2 ± 0.2	—	—	—	—

### Dissection of the Different β-Catenin-binding Regions of APC Repeat 3

We next looked at APC R3 constructs comprising each of the three different β-catenin binding interfaces either alone or in combination (Figure 1B), and the affinities were measured by fluorescence anisotropy using APC labelled at residue 1,501 with fluorescein (Figure 4; Table 1). Domains b and c could be expressed in isolation, but domain a could not. The binding of the phosphodomain c was only detectable in the phosphomimetic form, p-c (*K*_d_ of 160 nM), suggesting that this region only interacts with β-catenin in the cell when phosphorylated, which is consistent with what is observed in the crystal structures (as discussed above)*.* The lysine-binding domain b has a dissociation constant of 246 nM, which is similar to that of p-c; when domain b is combined with unphosphorylated domain c (bc), the affinity shows only a small increase (196 nM) relative to b alone, again consistent with the weak interaction of unphosphorylated c with β-catenin. When domain b is combined with domain a (ab) the affinity increases approximately 2.5-fold relative to b alone (90 nM versus 246 nM). The entire unphosphorylated APC fragment comprising all three domains (abc) has a 12-fold higher affinity than bc and a 5-fold higher affinity than ab. The increase in affinity upon adding unphosphorylated c to ab is somewhat surprising given the very small increase in affinity observed upon adding unphosphorylated c to b. Adding p-c to ab (i.e., the entire phosphorylated APC fragment, p-abc) results in the largest increase in affinity of adding any single domain (∼18-fold, 5.3 nM versus 90 nM).

**FIGURE 5:**
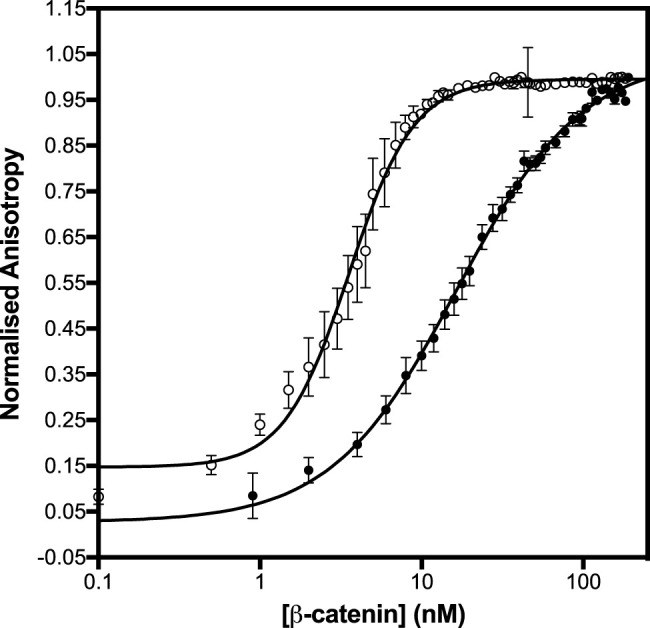
Binding of β-catenin to the unphosphorylated and phosphomimetic forms of APC_abc_. The dissociation constants were measured by fluorescence anisotropy using APC_abc_ labelled with fluorescein at Cys 1501. The unphosphorylated APC_abc_ is shown in black circles and the phosphomimetic, APC_abc_, in open circles. The data are shown as the mean ± SEM of at least three replicates.

Differences were observed in the shapes of the binding curves, indicating that there is cooperative binding of the subdomains for some of the APC constructs but not others. Cooperativity was observed for pAPC, as indicated by a Hill coefficient of 2, but not for the unphosphorylated APC or for any of the phosphorylated or unphosphorylated fragments (Hill coefficient of 1 within error) (Figure 5 and Table 1). This result suggests that the binding of the phosphorylated domain c results in an increase in affinity of the other domains, presumably due to the tethering of one domain bringing the other domains into proximity.

Because the lysine-binding domain b is the middle domain, it cannot be deleted in the same way as the helical domain a and phospho domain c. However, its affinity for β-catenin can be dramatically weakened by mutation of the lysine-interacting residues D1486 and E1494 (Xing et al., 2003; Ha et al., 2004; Kohler et al., 2008). The mutation D1486S lowered the affinity of unphosphorylated APC by more than 100-fold (2 μM vs 16 nM). (E1494T reduces the affinity to below the detection limit of the experiment (>5 μM)). This result suggests that domain a cannot bind to β-catenin in the absence of sufficient contacts made by domains b and c. D1486S lowered the affinity of pAPC 7.5-fold (30 nM vs. 5 nM). The affinity of pAPC_abc_ D1486S is higher than the affinity of p-c alone (160 nM), suggesting that some contacts of domain a with β-catenin are retained when domain c is bound; alternatively there may be some fraying of the β-catenin interface in the isolated p-c fragment, meaning that p-c does not effectively recapitulate the interactions present in the full p-abc and p-abc D1486S constructs.

We also investigated the contributions of the three domains of APC to the kinetics of β-catenin binding by stopped-flow fluorescence measurements of the fluorescein-labelled APC (Figure 5). (The kinetics of abc were also measured using Alexa 488-labelled APC, and similar kinetics were observed to those obtained using fluorescein-labelled APC). Two phases could be detected in the dissociation kinetics, a fast phase (∼35% amplitude) and a slow phase (∼65%). Deletion of domains a and c (constructs bc and ab, respectively) results in small (∼3-fold) decreases in the association rate and small (2-fold) increases in the dissociation rate (Table 1; Figure 4). Only one dissociation phase could be detected for bc, but given that for many of the variants there is a relatively small difference in the rates of the two phases, it could be that they are too similar in bc to distinguish. The introduction of the phosphomimetics (p-abc), which strengthens the interaction of domain c with β-catenin, has a small effect on the association kinetics (4-fold) and a larger effect on the dissociation kinetics than on the association kinetics, decreasing the fast dissociation phase 6-fold and the slow dissociation phase 50-fold. In contrast, the introduction of the D1486S mutation, which weakens the interaction of domain b with β-catenin, has negligible effect on the association kinetics of p-abc but a large effect on the dissociation kinetics, decreasing the fast phase 40-fold and the slow phase 200-fold. The association and dissociation kinetics of (unphosphorylated) abc D1486S could not be measured due to the very low binding affinity of this construct.

### Modulation of the Binding Interfaces of APC by Linker Insertions

**FIGURE 6:**
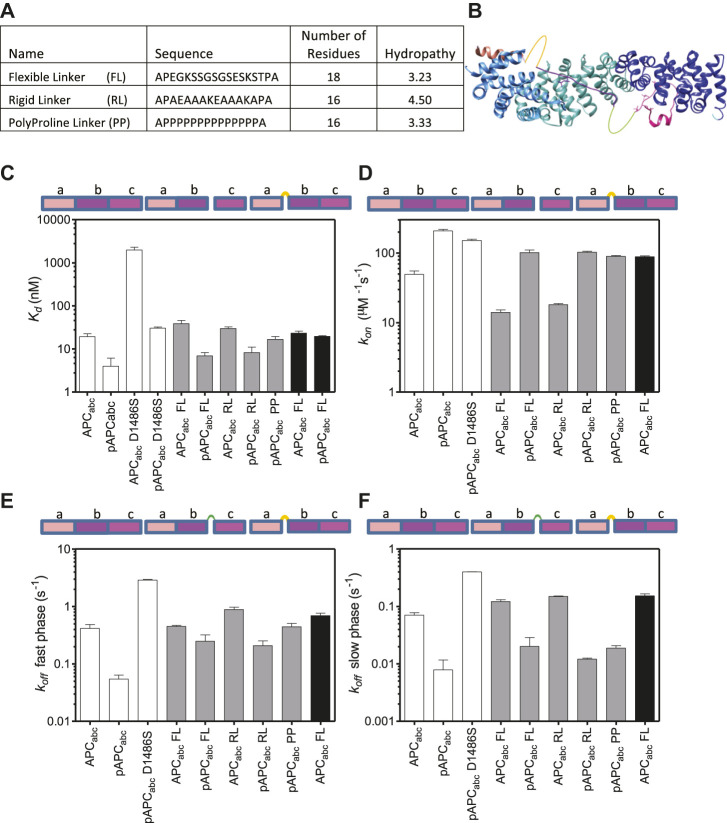
The effect of linker insertions and mutation of the lysine-interaction residue D1486S in APC on β-catenin-binding. **(A)** The sequences and properties of the linkers inserted into APC_abc_. The hydropathy was calculated using mean hydropathy of a sequence (Kyte and Doolittle, 1982). **(B)** Schematic showing the two sites of linker insertions in APC. **(C)** Dissociation constants were measured by fluorescence anisotropy of the APC variants labelled with fluorescein at Cys 1501, and the data are shown as the mean ± SEM of three replicates. **(D)** The association rates and the rates of the two dissociation phases, fast **(E)** and slow **(F)**, were measured by stopped-flow fluorescence. The data shown are the mean ± SEM of at least two replicate experiments consisting of five kinetic traces at each concentration measured.

To further probe the relationships between the three binding interfaces within APC, linkers were inserted at two different sites where there are no visible contacts with β-catenin in the crystal structures (Ha et al., 2004; Xing et al., 2004) (Figures 2, 6). The first site was between domains a and b (between residues 1,478 and 1,479) at the C-terminus of the α-helix. The second site was between domains b and c (between residues 1,500 and 1,501). This region of APC is four amino acids in length and is not visible in the crystal structure of the complex, suggesting that it is highly dynamic. Three types of linkers were used: one flexible linker and two rigid linkers (Figure 6). The linkers were all designed to be of similar length (16–19 amino acids) and have similar charge properties. The flexible linker (FL) is based on the (SG)_4_ linker between tandem immunoglobulin domains but also includes lysine and glutamate residues to aid solubility (Chen et al., 2013). The rigid linker RL forms an α-helix stabilised by glutamate-lysine salt bridges (Marqusee and Baldwin, 1987; Arai, 2021). The polyproline linker forms an extended left-handed helix and is regarded as a rigid “molecular ruler” (Schuler et al., 2005; Adzhubei et al., 2013). This linker is expected to be longer (∼4 nm) than the RL linker (∼1.8 nm).

Insertion of either the rigid or the flexible linker at 1,500 between domains b and c into either APC_abc_ or pAPC_abc_ reduced the affinity ∼1.5–2-fold indicating that most of the contacts with β-catenin were not disrupted (Table 1; Figure 6). A reduction in affinity even without any disruption of contacts is expected due to the greater entropy cost of closing the longer loop formed by the linker upon binding to β-catenin. Insertion of the polyproline linker into pAPC_abc_ reduced the affinity three-fold (16.4 nM versus 5.3 nM). The affinity was similar to that of unphosphorylated APC_abc_ (16.5 nM) that has a weakened c domain and pAPC_abc_ D1486S (30 nM) that has a weakened b domain, suggesting that the polyproline linker disrupts the interface and prevents domains b and c either side of it from binding to β-catenin simultaneously. Only the flexible linker was inserted at 1,478 (between domains a and b), and it did not change the binding affinity of APC_abc_ but reduced the affinity of pAPC_abc_ 5-fold, suggesting disruption of contacts in domains a and/or b.

As observed at equilibrium, the linker insertions at 1,500 (between b and c) had only small effects on the association rate, decreasing it ∼2-fold for both APC_abc_ and pAPC_abc_. There were also small increases (∼2.5-fold) in the dissociation rates. Insertion of the polyproline linker into pAPC_abc_ had a larger effect on the dissociation kinetics, increasing the rate of the fast dissociation phase 6-fold to a value similar to that of APC; the rate of the slow dissociation phase was increased 9-fold but it was still much slower than constructs APC_abc_ and pAPC_abc_ D1486S with weakened c/b domain. Interestingly, insertion of the flexible linker at 1,478 (between a and b) increased the association rate of APC_abc_ 2-fold. This result could be due to some steric stress between binding interfaces of the two domains that is relieved upon linker insertion.

The Hill coefficient was ∼1 for APC_abc_ with either the flexible or rigid linker inserted at bc, indicating a single binding event. In contrast, for pAPC_abc_ with the flexible or rigid linker inserted at the same position the Hill coefficient was ∼2, indicating that cooperativity between b and c is maintained upon linker insertion. Insertion of the polyproline linker into bc in pAPC_abc_ gave a Hill coefficient of 1, consistent with this linker preventing the binding of b and p-c simultaneously. From these results and the results for the truncations showing that the Hill coefficient is 1 for constructs ab and abc lacking the high-affinity p-c domain, we can conclude that a and b are not independent domains but rather form one contiguous binding surface.

## Discussion

**FIGURE 7:**
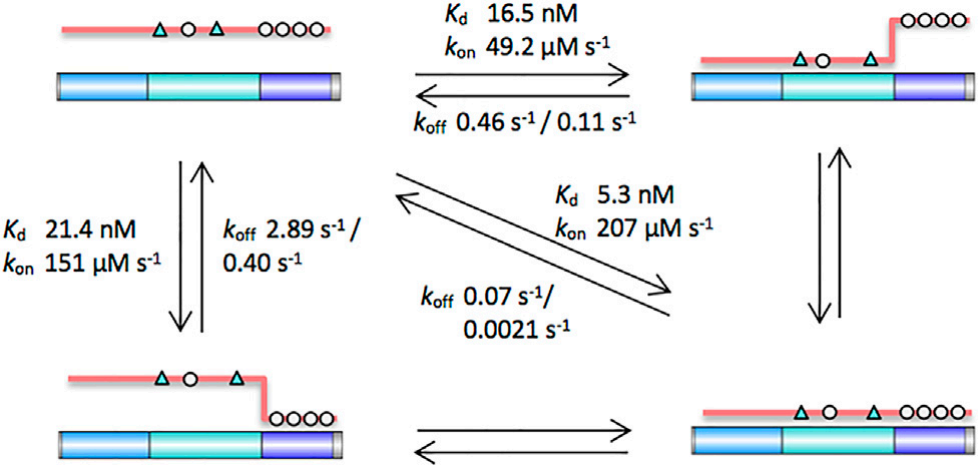
Schematic of the contributions of the different subdomains of APC to the interaction with β-catenin. The key contacting residues are denoted in triangles (lysine-binding residues) and circles (phosphomimetics). Key dissociation constants and rates of association and dissociation are shown. The intrinsic β-catenin-binding affinity of the b domain can be estimated from the affinity of APC, which has a weakened c domain. The intrinsic β-catenin-binding affinity of the c domain can be estimated from the affinity of pAPC D1486S, which has a weakened b domain.

Here we explore the molecular recognition of the third 20aa repeat region of APC as an example of the β-catenin binding partners that are phospho-regulated in the β-catenin destruction complex (BDC). In its unphosphorylated form, APC contacts are predominantly with β-catenin ARM5-9, thus allowing the interaction of β-catenin ARM1-4 with axin (Ha et al., 2004). The formation of the trimeric complex of β-catenin, APC and axin has been shown to lead to increased phosphorylation of β-catenin, thereby promoting its recognition and ubiquitination by the E3 ligase SCF^β−TrCP^ (Ranes et al., 2021). Phosphorylated APC binds across all twelve of β-catenin’s ARM repeats, thereby displacing axin, retaining β-catenin in the cytoplasm and restarting the process of interactions in the BDC (Schaefer and Peifer, 2019). APC repeat 3 can be divided into three subdomains, and we used a combination of single-site substitutions, truncations and insertions to dissect the contributions of the subdomains to the interaction. As the subdomains are not fully separate from each other there may be fraying of the interface formed by one domain when its adjacent domain is absent, and consequently there are some caveats in the interpretation of the effects of the variants where we have truncated one or more of the subdomains. Nevertheless, the following conclusions can be made (Figure 7):

1) In a multivalent system such as this, the strength of the interactions of the individual subdomains are context dependent, i.e., they are dependent on the presence or absence of the other subdomains. The most striking examples are the effects of a mutation that disrupts key contacts in domain b and the effects of mutations that mimic phosphorylated residues in domain c: The mutation D1486S in domain b causes a 100-fold decrease in the affinity of the unphosphorylated APC, whereas there is only a 4-fold decrease in the affinity when the mutation is made in the phosphomimetic pAPC. Likewise, the phosphomimetic mutations in domain c cause a 100-fold increase in the affinity of APC D1486S, whereas there is only a 3-fold increase in the affinity when the same phosphomimetic mutations are introduced in APC.

2) This cooperativity of the interactions is evident also in the different shapes of the binding curves, and it can be quantified with the Hill coefficient. Cooperativity (Hill coefficient of 2) is only observed for pAPC and for loop insertions where b and c retain their ability to bind simultaneously. No cooperativity (Hill coefficient of 1) is observed for variants in which either b or c has a weakened affinity (APC, pAPC D1486S), for insertions such as polyproline linker where b and c cannot bind simultaneously, and for the fragment ab lacking domain c. The Hill coefficient of 1 observed for constructs ab and abc indicates that a and b effectively form a contiguous domain rather than two independent domains.

3) The similar affinities of pAPC D1486S and APC, and of fragments p-c and b, suggest that the interactions made by these two domains with β-catenin are of similar strength (Figure 6). We expect the affinity of phosphorylated c domain to be higher than our phosphomimetic c domain, and therefore it is likely that in the cell p-c makes a greater contribution than b to the interaction of pAPC with β-catenin.

4) In a multivalent system, large effects of domain deletion/mutation on the dissociation kinetics are expected due to the probability of rebinding being much lower when one or other of the domains is absent or mutant. A single phase is observed for the association kinetics of APC and all of the variants, whereas the dissociation kinetics are biphasic. Several different kinetic mechanisms can be envisaged: Binding/unbinding of the different domains of APC could be sequential or concurrent. Further, given that the affinities of domains b and c are similar, there could also be parallel (alternative) binding/unbinding pathways of similar energies corresponding to different orders of domain binding/unbinding. This is analogous to the parallel (un)folding pathways observed for repeat proteins, which is a result of the different repeats having similar stabilities (Lowe and Itzhaki, 2007; Tripp and Barrick, 2008; Werbeck et al., 2008; Tsytlonok et al., 2013; Aksel and Barrick, 2014; Hutton et al., 2015). The mutations affect both of the dissociation phases rather than only one or the other. The linker insertions also affect both phases, and the largest effects are observed for the polyproline linker (PP) which prevents b and c from binding simultaneously. Despite mutation of the phosphorylated residues in domain c (pAPC vs. APC) and mutation of the lysine-binding residues in domain b (pAPC vs. pAPC D1486S) having similar effects on the equilibrium binding affinity, mutation of domain b has a larger effect than mutation of domain c on the dissociation kinetics. In the association kinetics, it is mutation of domain c that has the larger effect. Insertion of random or flexible linkers between b and c slows down the association and speeds up the dissociation, as expected. Insertion of the polyproline linker (PP) is the most disruptive and prevents simultaneous binding of b and c domains and ablates the tethering effect, as shown by the similarity in the binding affinity of pAPC PP to those of APC and pAPC D1486S. The fast dissociation rate of pAPC PP is similar to that of APC, but its slow dissociation rate is in between those of APC (0.11 s^−1^) and pAPC (0.0021 s^−1^), and its association rate is in between those of APC (49 μM^−1^s^−1^) and pAPC (207 μM^−1^s^−1^). The observation that the mutations and insertions affect both dissociation phases rather than only one or other indicates that there is not a simple sequential unbinding mechanism and that the dissociation of b and c domains is tightly coupled.

We can compare these findings with those of another β-catenin ligand, TCF7L2 (Smith et al., 2021). Like APC, TCF7L2 has a b domain with lysine-binding residues. However, unlike in APC, the interaction of the b domain of TCF7L2 with β-catenin is fuzzy, and it does not adopt a fixed conformation and is structurally malleable. Likely as a result of this fuzzy interaction. Like β-catenin-APC, the kinetics of the β-catenin-TCF7L2 dissociation and association are complex. However, unlike β-catenin-APC, a sequential mechanism of domain binding/unbinding can be readily discerned for β-catenin-TCF7L2 as well as a shift to an alternative pathway upon mutation in which the order of domain binding/unbinding is reversed.

Above, we compare the effects of the different APC variants on the binding affinity in terms of how they disrupt the interaction with β-catenin. However, these mutations (which include charge substitutions and linker insertions) could potentially alter the binding affinity by changing the conformational ensemble of the unbound APC. To address this point, we measured the CD spectra of a subset of the APC constructs and compared them with literature values for folded, (pre)molten globule and unfolded proteins from the Protein Circular Dichroism Data Bank (PCDDB) (Supplementary Figure S6). We found that the spectra were similar to each other and random coil in nature, suggesting that the unbound APC ensemble is not significantly altered by the mutations made.

It is important to understand kinetic stability, as measured by dissociation rates, as well as thermodynamic stability in efforts to develop inhibitors of protein-protein interactions. β-catenin is upregulated in many cancers, but the identification of small molecule inhibitors is challenging given the very large, extended interaction surface. Moreover, positive and negative regulators share part of this interface, making selective inhibition even more difficult, and inhibitors must also avoid disrupting the interaction of β-catenin with E-cadherin at the cell membrane. An alternative approach is to direct the cytosolic pool of β-catenin for degradation using either small molecule PROTACs (proteolysis-targeting chimeras) or peptide-based molecules. To this end, dissecting the contributions of the different APC subdomains of APC to β-catenin binding will provide critical information for developing ligands capable of effectively engaging β-catenin.

## Data Availability

The raw data supporting the conclusion of this article will be made available by the authors, without undue reservation.
