# Supplementary material for: Multivalent Interaction of Beta-Catenin With its Intrinsically Disordered Binding Partner Adenomatous Polyposis Coli
Source: Front Mol Biosci. 2022 Jun 8;9:896493. doi: 10.3389/fmolb.2022.896493 (PMC9214244; doi:10.3389/fmolb.2022.896493)
Supplement: Supplementary file 1 [file DataSheet1.docx]

**Rowling et al.**

**Supplementary Information**


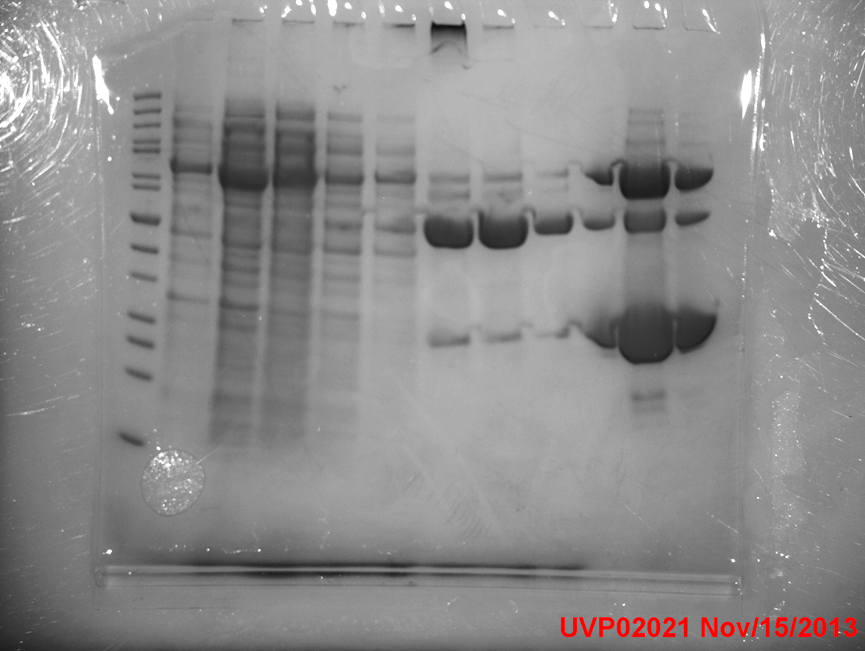


200

150

120

100

85

70

60

50

40

30

25

20

15

10

kDa

Gel 1

Lane 1 2 3 4 5 6 7 8 9 10 11


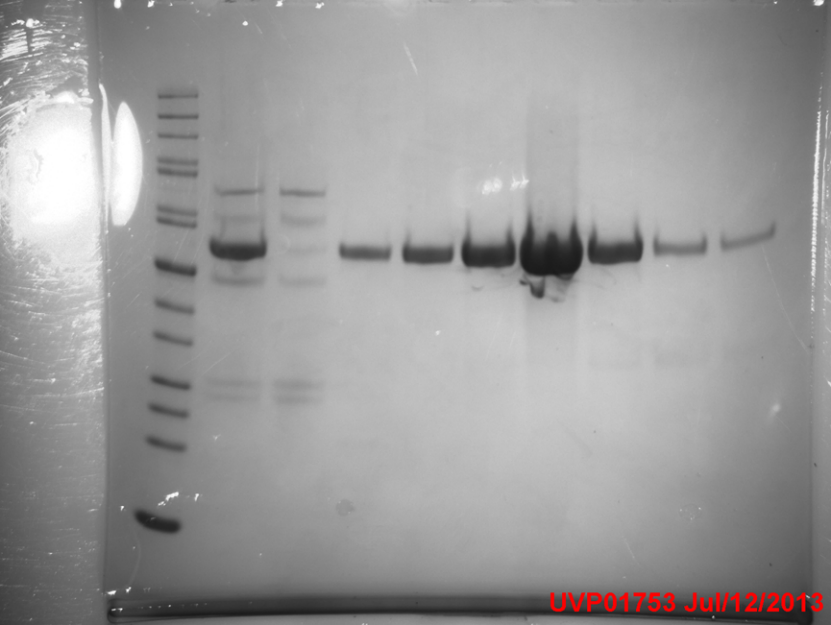


Gel 2

Lane 1 2 3 4 5 6 7 8 9 10

200

150

120

100

85

70

60

50

40

30

25

20

15

10

kDa

**Figure S1: Representation example of purification of** **β-catenin.** β-catenin was purified as described in the Methods and Materials**.** The proteins were separated on a 4-12 % Bis-Tris NuPAGE gel (Thermo Scientific). Gel 1 Lane 1: molecular weight markers, Lane 2: total protein post cell lysis, Lane 3: soluble protein post lysis, Lanes 4-6: unbound proteins eluting from the glutathione agarose resin, Lanes 7-10: post cleavage of GST-tag by thrombin and eluted from matrix, Lane 11: glutathione-agarose resin post cleavage of GST-tag and elution of β-catenin. Gel 2 Purification by ion-exchange chromatography Lane 1: molecular weight markers, Lane 2: pooled elution from glutathione agarose column, Lane 3: unbound material, Lanes 4-10: β-catenin elution, these fractions were pooled as the purified β-catenin, the identity confirmed by MALDI mass spectrometry.

**
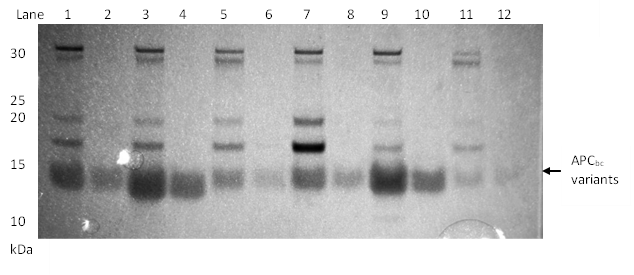
**

**Figure S2**: **Representative examples of purification of APC R3 variants.** Proteins are arranged in pairs: odd-numbered lanes are post glutathione-agarose affinity purification and proteolytic removal of GST-tag with thrombin; even-numbered lanes are post differential filtration purification and show the purified proteins used in experiments. The masses of the purified proteins were confirmed by MALDI mass spectrometry. Lanes 1 & 2: APC_ab_S2S4, Lanes 3 & 4: APC_ab_S3S4, Lanes 5 & 6: APC_ab_S1S2S3, Lanes 7 & 8: APC_ab_S1S2S4, Lanes 9 & 10 APC_ab_S1S3S4, Lanes 11 & 12: APC_ab_S2S3S4.

**(A)**

**(B)**

**(C)**

**Figure S3**: **Representative examples of competition fluorescence** **assays for phosphomimetic APC_bc_ variants binding to β-catenin.** Binding was measured with a competitive fluorescence assay using APC_bc_ labelled with Alexa-488 at Cys 1501. Glutamate substitutions were made at phosphorylation sites T1487E (T1), S1504E (S1), S1505E (S2), S1507E (S3), S1510E (S4) of APC_bc_ 20 nM Alexa-488-labelled APC_bc_ was incubated with 200 nM β-catenin at 25 ^o^C for 30 min prior to titration of the unlabelled APC_bc_ phosphomimetics. Experiments were performed in PBS buffer 1 mM DTT at 25 ^◦^C.

**(A)**

**(B)**

**Figure S4**: **Representative examples of fluorescence anisotropy assays for β-catenin binding to APC constructs** **labelled on Cys 1501 with fluorescein. (A)** APC_ab_; **(B)** p-APC_c_. β-catenin was titrated into 10 nM fluorescein-labelled APC constructs, and the anisotropy and fluorescence intensity were measured. There was a change in fluorescence intensity upon binding, and the anisotropy data shown have been corrected for these changes. Experiments were performed in PBS buffer 1 mM DTT at 25 ^◦^C.

**(B)**

**(C)**

**(A)**

**(D)**

**(E)**

**Figure S5: Representative examples of stopped-flow fluorescence traces for the association and dissociation of APC_abc_ and β-catenin.** Time-dependent change in fluorescence intensity upon rapid mixing of 10 nM fluorescein-labelled APC_abc_ and β-catenin at 200 nM **(A**), 400 nM **(B)**, and 600 nM **(C)**. The data from ~5 traces were averaged and fitted to a single-exponential function, and the residuals are shown below the main plot. **(D)** Time-dependent change in fluorescence intensity for the dissociation of the APC_abc_– β-catenin complex. 200 nM fluorescein-labelled APC_abc_–β-catenin complex was mixed with 10-times molar excess of unlabelled APC_abc_. The data from ~5 traces were averaged and fitted to the sum of two exponential phases, and the residuals are plotted below. **(E)** The observed rate constant was plotted as a function of the β-catenin concentration, from which the association rate constant, *k*_on_, was calculated. The errors bars are the standard deviations of the fits. Experiments were performed in PBS buffer 1 mM DTT at 15 ^◦^C.

**(A)**

**(B)**


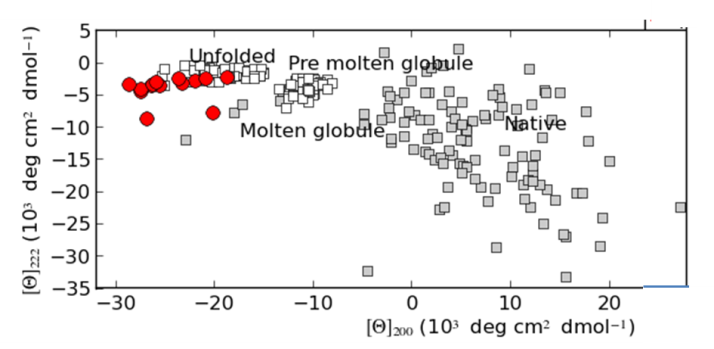


**Figure 6S: Secondary structural assessment of** **APC R3** **variants. (A)** The CD spectra of APC proteins in PBS buffer containing 1 mM DTE were measured at 25^o^C using a Chirasan CD (Applied Photophysics). All constructs showed similar spectra indicative of the majority of the structure being random coil. The spectra of the two APC_c_ fragments (APC_c_ and pAPC_c_ – without and with the phosphomimetic mutations) suggest that they have a slightly more ordered structure. **(B)** Uversky (2002) showed by plotting mean residue ellipticity at 222 nm versus mean residue ellipticity at 200 nm that disordered proteins (labelled ‘Unfolded’) fall into a distinct cluster compared with folded and molten globule proteins from the Protein Circular Dichroism Data Bank (PCDDB, white and grey squares). The data for the APC R3 variants (red symbols) fall into the unfolded region of the graph, indicating that they are intrinsically disordered. The plot was made using the Capito program (<https://data.nmr.uni-jena.de/capito/index.php> (Weidemann (2013)).

**References**

Uversky V. N. (2002). Natively unfolded proteins: a point where biology waits for physics. *Prot. Sci.* 11, 739–756. doi.org/10.1110/ps.4210102

Whitmore, L., Miles, A.J., Mavridis, L., Janes, R.W. and Wallace, B.A., (2017) PCDDB: new developments at the Protein Circular Dichroism Data Bank.*Nuc. Acids Res.* 45 (D1): D303-D307.  doi: 10.1093/nar/gkw796

Wiedemann, C., Bellstedt, P., Görlach, M. (2013). CAPITO - A web server based analysis and plotting tool for circular dichroism data. *Bioinformatics* 29, 1750-1757. [doi:org/10.1093/bioinformatics/btt278](https://doi.org/10.1093/bioinformatics/btt278).
